# Subacute Thyroiditis Presenting as a Painful Thyroid Nodule and Subclinical Hyperthyroidism: A Case Report

**DOI:** 10.7759/cureus.68886

**Published:** 2024-09-07

**Authors:** Merlin Perez Navarro, Diane Krieger

**Affiliations:** 1 Medicine, Florida International University, Herbert Wertheim College of Medicine, Miami, USA; 2 Endocrinology, Endocrinology Associates, Professional Association, Miami, USA

**Keywords:** anterior neck pain, de quervain thyroiditis, hyperthyroidism, subacute thyroiditis, ultrasound

## Abstract

This case report details the evaluation and management of a 55-year-old woman who presented to our endocrinology clinic due to low TSH and thyroid nodules previously evaluated by her ENT. The patient originally presented with anterior neck pain and dysphagia. Ultrasonography demonstrated thyroid nodules with suspicious features, prompting a fine needle aspiration (FNA). A biopsy showed a follicular lesion/atypia of undetermined significance (Bethesda III). Due to concerns for malignancy, a right lobectomy was recommended. Thyroid function tests showed subclinical hyperthyroidism. She presented to our endocrinology clinic and was diagnosed with subacute thyroiditis based on signs of symptoms, radioactive iodine scanning, and biochemical studies (elevated ESR). Within approximately seven months, thyroid function tests and inflammatory markers returned to baseline, and symptoms and physical findings resolved. The case highlights the importance of understanding the similarities and differences between subacute thyroiditis and malignant pathologies in order to avoid misdiagnosis and unnecessary procedures.

## Introduction

Subacute thyroiditis, also known as de Quervain thyroiditis or subacute granulomatous thyroiditis, is an inflammatory condition of the thyroid that commonly presents after viral infections, commonly influenza and adenovirus [1]. Typical clinical features include jaw pain, asymmetric or diffuse painful and firm goiter, fever, and signs and symptoms of hyperthyroidism followed by those of hypothyroidism. While most patients have bilateral symptoms, those with “creeping thyroiditis” can start with symptoms on one side, which progress to the other in days or weeks [2]. The diagnosis can be made based on the medical history, physical exam, lab result, and radioactive iodine uptake scan. Thyroid function tests during the thyrotoxic phase usually demonstrate increased T3 and T4 and decreased TSH. This is followed by a brief normalization of thyroid function, which is followed by a hypothyroid phase with decreased T3 and T4 and increased TSH. Confirmatory test results include elevated ESR and C-reactive protein (CRP) along with decreased iodine uptake shown in a radioiodine uptake study [1]. The disease is self-limiting in most cases and resolves within weeks to months. Patients can experience relapses, and permanent hypothyroidism occurs in less than 20% of cases [1].

## Case presentation

**Figure 1:**
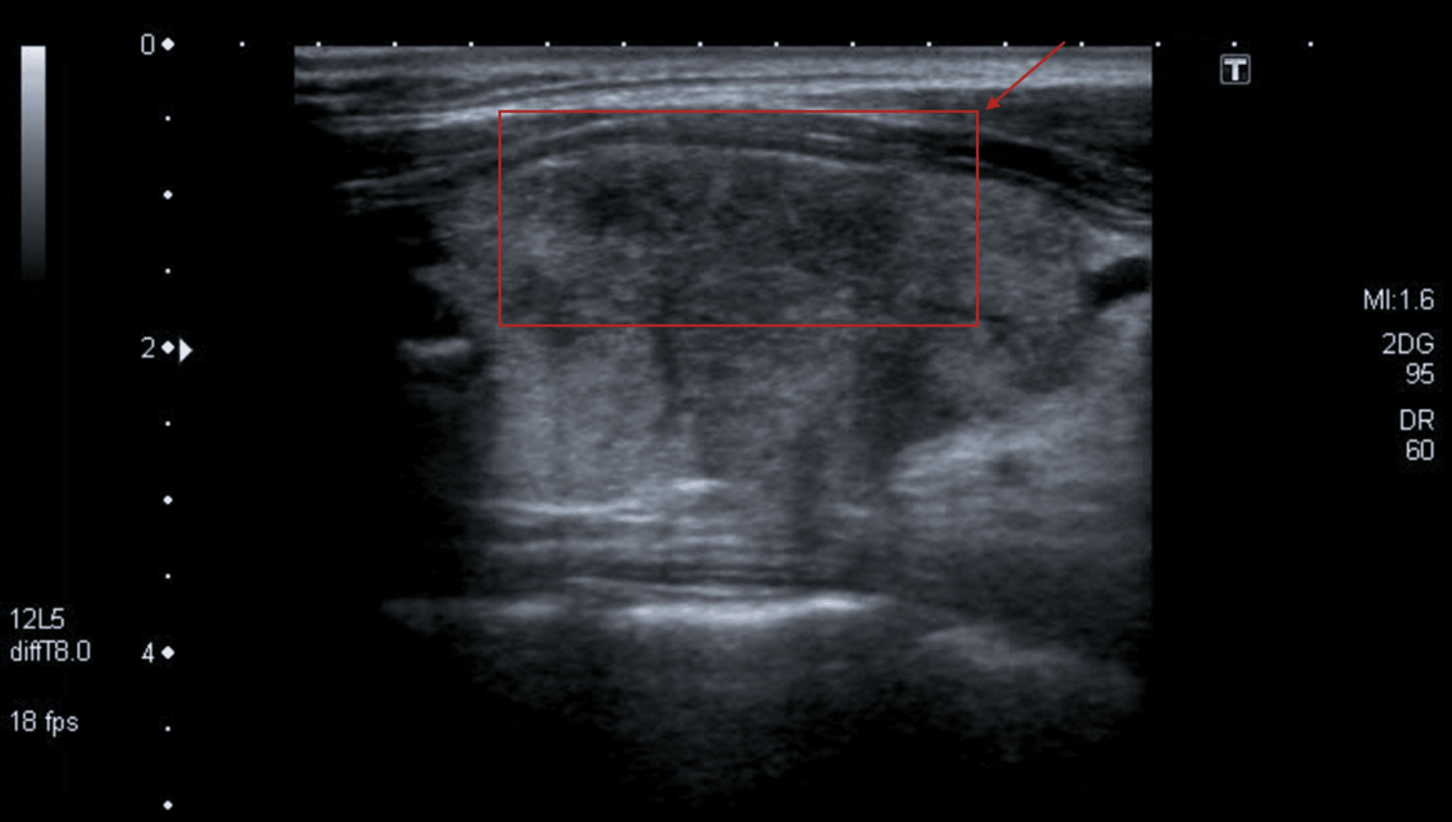
Ultrasound findings in subacute thyroiditis: confluent, hypoechoic region in thyroid parenchyma

A 55-year-old female with no significant past medical history self-referred to endocrinology due to thyroid nodules previously evaluated by an ENT specialist. The patient initially presented to the ENT specialist due to dysphagia and anterior neck pain that had started about a month before. Labs ordered at the first visit were remarkable for a low TSH of 0.01 uIU/mL and a free T4 index of 3.8 (normal range 1.4-3.8) (Table 1). A thyroid ultrasound was performed and revealed that the right thyroid lobe measured 5.4 × 2.4 x 2.7 cm and was mostly replaced by a solid heterogeneous nodule with microcalcifications measuring 4.1 x 2.4 x 2.2 cm (rated as Thyroid Imaging Reporting and Data System (TI-RADS) 5). The left lobe measured 4.1 x 1.3 x 1.8 cm with normal echo texture and unremarkable vascularity; there was a complex hypoechoic nodule within the upper pole measuring cysts 0.6 x 0.4 x 0.3 cm (TI-RADS 4) with no abnormal calcifications (Figure 1). A fine needle aspiration (FNA) of the right nodule demonstrated atypia of undetermined significance (Bethesda III). A repeat FNA 10 days later also was interpreted as Bethesda III. A thyroid surgery was recommended.

**Table 1:** Lab values throughout the course of subacute thyroiditis

Date	TSH (uIU/mL) - normal: 0.5-5	Free T4 (ng/dL) - normal: 0.8-1.9 or FT4 index - normal: 1.4-3.8	T3 (ng/dL) - normal 60-180	ESR (mm/hour) - normal <20
1/19/24	0.01 (low)	3.8 (Ft4I)	-	-
2/8/24	0.148 (low)	1.24	120	34
3/14/24	0.087 (low)	1.34	127	49 (high)
5/1/24	5.940 (high)	1.09	91	-
7/1/24	3.300	0.94	71	5

The patient denied a personal or family history of thyroid cancer but did mention a history of hypothyroidism in her mother. The patient was referred to a second ENT specialist who repeated the FNA of the right nodule (Bethesda II) but agreed with the plan for surgery due to the size of the nodule. The patient then presented to our clinic for further evaluation. A thorough history revealed that the patient was experiencing anterior neck pain radiating to the ear, which had moved from the right neck to the left neck over several weeks. She also endorsed occasional tremors and night sweats, recent weight loss, and fatigue, which were resolving. The patient denied childhood head and neck radiation exposure. Due to our suspicion of an alternate diagnosis of subacute thyroiditis, we ordered repeat thyroid function tests, which again showed a subclinical hyperthyroid profile (low TSH with normal free T4 and total T3) with a high normal ESR (Table 1).

To confirm the diagnosis, a radioactive iodine uptake scan was performed and revealed diffusely decreased iodine uptake of less than 1%, consistent with subacute thyroiditis. Acetaminophen was sufficient for pain management. Genomic sequencing of the second right thyroid FNA was obtained and determined a very low risk of malignancy. The patient was informed about the natural history of subacute thyroiditis and that surgery was not indicated. Four weeks later, the pain persisted and labs showed that the patient was still in the subclinical hyperthyroid phase and ESR was increased, without significant hyperthyroid symptoms. Three months after she presented to endocrinology, her labs showed a transition to the hypothyroid phase, which was asymptomatic. At five months of follow-up, thyroid function values had returned to her euthyroid baseline and ESR had normalized (Table 1). The right thyroid nodule was no longer palpable. Her weight was stable, energy was normal, and anterior neck pain had resolved. She was advised to repeat a thyroid ultrasound.

## Discussion

Subacute thyroiditis is an inflammatory condition commonly preceded by infections, namely those caused by the mumps virus, coxsackie virus, influenza virus, echovirus, and adenovirus. It has a typical clinical presentation that includes neck pain with or without radiation to the ear, throat, or jaw, low-grade fever, fatigue, and signs and symptoms of hyperthyroidism. The condition usually manifests with a triphasic response, progressing from hyperthyroidism through euthyroidism to hypothyroidism and finally returning to the euthyroid state. The thyrotoxic phase tends to last from two to eight weeks and is caused by damage to follicular cells, which leads to the release of stored thyroid hormones [3]. The hypothyroid phase has a similar duration, although it can be permanent in approximately less than 20% of cases. This phase is caused by the depletion of pre-formed thyroid hormones and impaired synthesis of new hormones as a result of follicular cell destruction [3]. The condition is clinically diagnosed based on history, symptoms, and biochemical evaluations. Obtaining a thorough medical history is an essential component of correctly diagnosing subacute thyroiditis as certain clues, including radiating neck pain and symptoms of hyperthyroidism, can point to this condition. In this case, the diagnosis was missed by two different physicians, possibly due to gaps in history taking and a premature shifting focus to features concerning for a malignant process. Similar cases of misdiagnosis of subacute thyroiditis have been previously reported, indicating the complexity of diagnostic differentiation and the importance of a thorough history [4]. 

The diagnostic workup of subacute thyroiditis includes thyroid function tests, inflammatory markers such as CRP and ESR, and a radioactive iodine uptake scan. Thyroid function values vary depending on the disease stage. It is important to note that they can also be within normal limits, just as free T4 and T3 remained normal throughout the course of the condition in this patient [1]. CRP and ESR, especially the latter, are typically elevated due to the inflammatory nature of subacute thyroiditis. An ultrasound is not required to make the diagnosis nor is it routinely performed. It is occasionally performed to rule out malignant pseudothyroiditis (thyroid pain caused by thyroid carcinoma or thyroid lymphoma). Similar to subacute thyroiditis, malignant pseudothyroiditis can present with a painful goiter, signs of hyperthyroidism, and increased inflammatory markers such as CRP [5]. Thyrotoxicosis may be present and involves the same pathophysiology as that seen in subacute thyroiditis; infiltration of the thyroid by malignant cells leads to lysis of thyrocytes and subsequent release of thyroid hormones [5].

Ultrasound is essential in differentiating malignant pseudothyroiditis and subacute thyroiditis. Findings in subacute thyroiditis are commonly described as focal or multifocal lesions with poorly defined hypoechoic echogenicity [6]. Malignant pseudothyroiditis has similar sonographic features, but may also present with irregular and spiculated margins and homogeneous lymphadenopathies [5]. An FNA is not generally required but can be obtained in cases of concern for neoplasm or infection. In subacute thyroiditis, an FNA typically shows multiple multinuclear giant cells with fragments of inflammatory cells and follicular epithelial cells [7]. It is important to highlight that if the FNA is reported as anything other than Bethesda II, this may lead to unnecessary repeated FNAs or surgery, which was the case with our patient. Decreased radioactive iodine uptake scan is classically seen in subacute thyroiditis and helps differentiate it from other hyperthyroid disorders such as Grave’s disease but not from other causes of thyroiditis [3]. The treatment for subacute thyroiditis is generally aimed at symptom management. During the thyrotoxic phase, beta-blockers can be used to control symptoms of hyperthyroidism. Pain and tenderness can be targeted with nonsteroidal anti-inflammatory drugs (NSAIDs), but in severe cases refractory to NSAIDs, corticosteroids can be used [8]. During the hypothyroid phase, levothyroxine may be used but at a low dose so that endogenous TSH levels are not suppressed, and the euthyroid state can be recovered [8].

## Conclusions

Subacute thyroiditis is a self-limiting inflammatory thyroid disorder that commonly follows an infectious process. It is diagnosed based on history and signs and symptoms, often accompanied by other diagnostic exams and biochemical markers. In patients with atypical presentations and/or concern for malignancy or infection, ultrasonography can be used for further evaluation. Subsequent use of an FNA, although rarely indicated for this condition, can be prompted by concerning sonographic features. This can trigger a cascade of unnecessary testing and procedures including surgery, as was recommended for our patient. In this case, the possibility of subacute thyroiditis as an explanation of the patient’s discomfort was overlooked by two physicians.

Thus, it is imperative for physicians to recognize that although subacute thyroiditis can present with suspicious nodular lesions on ultrasonography, other findings such as anterior neck pain and tenderness radiating to the ear and creeping symptoms from one side to the other typically favor the self-limiting diagnosis over that of malignancy. Other features include elevated inflammatory markers, thyroid function test disruptions, and diffusely decreased radioactive iodine uptake. It is possible that our patient’s thyroid function tests would have shown overt hyperthyroidism rather than subclinical hyperthyroidism if they had been checked earlier in her course. While the hyperthyroid phase typically lasts up to eight weeks, our patient’s lasted approximately twelve weeks. This case emphasizes the importance of good history taking and keeping a wide differential when formulating treatment plans.

## References

[1] Jameson J, Mandel SJ, Weetman AP (2024). Hyperthyroidism and other causes of thyrotoxicosis. In:. Harrison's Principles of Internal Medicine.

[2] Nakamura T, Kakimoto H, Morita S, Mizota M, Iwamoto J (2021). Subacute thyroiditis presenting with creeping in a 6-year-old boy. Clin Pediatr Endocrinol.

[3] Pearce EN, Farwell AP, Braverman LE (2003). Thyroiditis. N Engl J Med.

[4] Zeng W, Tan S, King TF (2022). Subacute thyroiditis presenting as a painful suspicious thyroid nodule. Endocrinol Diabetes Metab Case Rep.

[5] Ghanassia E, Monpeyssen H, Marchand JG, Coste T, Garrel R, Costes-Martineau V, Marcy PY (2023). Malignant pseudothyroiditis after COVID-19 infection revealing a poor-cellular variant of a thyroid anaplastic carcinoma. Preprints.

[6] Lee YJ, Kim DW (2016). Sonographic characteristics and interval changes of subacute thyroiditis. J Ultrasound Med.

[7] Ranganath R, Shaha MA, Xu B, Migliacci J, Ghossein R, Shaha AR (2016). de Quervain's thyroiditis: a review of experience with surgery. Am J Otolaryngol.

[8] Ray I, D'Souza B, Sarker P, Agarwal P (2022). Management of subacute thyroiditis - a systematic review of current treatment protocols. Int J Gen Med.

